# Simvastatin Promotes Cardiac Myocyte Relaxation in Association with Phosphorylation of Troponin I

**DOI:** 10.3389/fphar.2017.00203

**Published:** 2017-04-19

**Authors:** David A. MacDougall, Sara D. Pugh, Harpreet S. Bassi, Sabine Lotteau, Karen E. Porter, Sarah Calaghan

**Affiliations:** ^1^School of Biomedical Sciences, University of LeedsLeeds, UK; ^2^Leeds Institute of Cardiovascular and Metabolic Medicine, University of LeedsLeeds, UK

**Keywords:** statin, pleiotropy, cardiac, nitric oxide, caveolin, lusitropy, diastole

## Abstract

The number of people taking statins is set to increase across the globe due to recent changes in prescription guidelines. For example, half the US population over 40 is now eligible for these drugs, whether they have high serum cholesterol or not. With such development in policy comes a stronger need for understanding statins’ myriad of effects. Surprisingly little is known about possible direct actions of statins on cardiac myocytes, although claims of a direct myocardial toxicity have been made. Here, we determine the impact of simvastatin administration (40 mg/kg/day) for 2 weeks in normocholesterolemic rats on cardiac myocyte contractile function and identify an underlying mechanism. Under basal conditions, statin treatment increased the time to half (*t*_0.5_) relaxation without any effect on the magnitude of shortening, or the magnitude/kinetics of the [Ca^2+^]_i_ transient. Enhanced myocyte lusitropy could be explained by a corresponding increase in phosphorylation of troponin I (TnI) at Ser^23,24^. Statin treatment increased expression of eNOS and Ser^1177^ phosphorylated eNOS, decreased expression of the NOS-inhibitory proteins caveolins 1 and 3, and increased (*P* = 0.06) NO metabolites, consistent with enhanced NO production. It is well-established that NO stimulates protein kinase G, one of the effectors of TnI phosphorylation at Ser^23,24^. Trends for parallel changes in phospho-TnI, phospho-eNOS and caveolin 1 expression were seen in atrial muscle from patients taking statins. Our data are consistent with a mechanism whereby chronic statin treatment enhances TnI phosphorylation and myocyte lusitropy through increased NO bioavailability. We see no evidence of impaired function with statin treatment; the changes we document at the level of the cardiac myocyte should facilitate diastolic filling and cardiac performance.

## Introduction

Statins reduce cardiovascular morbidity and mortality and are widely prescribed to those at risk of cardiovascular disease, and with established heart disease. A recent reduction in the cardiovascular risk threshold for statin prescription across the globe means that the number of people taking statins is set to increase. For example, 13 million more individuals are now eligible for statin therapy in the US due to recent amendments to ACC/AHA guidelines (Pencina et al., 2014; Stone et al., 2014). When considered in combination with high-dose statin treatment regimens, which are recommended for some patient groups (e.g., Chan et al., 2007), the importance of fully understanding statins’ effects on the cardiovascular system is highlighted.

Statins inhibit the enzyme HMG CoA reductase which is the rate limiting step in cholesterol synthesis. However, it is well-established that the impact of statins on the cardiovascular system extends far beyond lowering serum LDL cholesterol. These ‘pleiotropic’ effects can be beneficial, and include increased NO bioavailability, anti-oxidant and anti-inflammatory actions (e.g., see, Davignon, 2004). Much research in this field has focused on endothelial cells. At the same time, a body of work has accumulated suggesting concurrent detrimental effects on the cardiovascular system, mediated through the same HMG CoA reductase pathway, including the promotion of coronary artery calcification (see, Okuyama et al., 2015). Surprisingly, little work has determined direct effects of statins on the cardiac myocyte, which is the focus of this work. It is essential that the full impact of statins is understood if prevention and treatment of cardiovascular disease are to be optimized.

Statins’ pleiotropic effects can be ascribed to depletion of cholesterol or other products of the HMG CoA reductase pathway. The latter include the isoprenoids farnesyl pyrophosphate and geranylgeranyl pyrophosphate which act to target small and heterotrimeric G proteins to the membrane (Goldstein and Brown, 1990), and coenzyme Q, a component of the mitochondrial respiratory chain, whose depletion has been linked with mitochondrial dysfunction and statin-induced ‘myocardial toxicity’ (Okuyama et al., 2015). For cholesterol-dependent pleiotropic effects, statins may initially alter tissue cholesterol levels by limiting *de novo* cholesterol synthesis. As cellular cholesterol homeostasis is maintained through sterol regulatory element (SRE) transcriptional regulation of proteins involved in the synthesis, uptake and efflux of cholesterol (Reboulleau et al., 2012), changes in tissue cholesterol are likely to be normalized over time. However, altered expression of cholesterol homeostatic proteins may have functional consequences which contribute to statins’ effects.

The influence of statins on NO provides an excellent illustration of their broad pleiotropic effects. Statins increase NO bioavailability by a number of isoprenoid-dependent mechanisms mediated through Rho/Ras which include increased eNOS expression (*via* stabilization of eNOS mRNA), enhanced eNOS activity (*via* enhanced Akt-dependent phosphorylation) and reduced NO scavenging (*via* NADPH oxidase activity) (Laufs and Liao, 1998; Urbich et al., 2002; Nakagami et al., 2003). In addition, statin treatment of endothelial cells and cardiac myocytes (*in vitro)* decreases expression of caveolin (Feron et al., 2001; Pugh et al., 2014), a protein which contributes to cholesterol efflux (Ikonen and Parton, 2000) and is under SRE transcriptional control (Bist et al., 1997). Caveolin is a key inhibitory regulator of eNOS and this cholesterol-dependent change in caveolin expression is associated with increased eNOS activity (Feron et al., 2001; Pugh et al., 2014). Thus isoprenoid- and cholesterol-dependent effects contribute to statin regulation of NO bioavailability.

The nature of cholesterol-SRE dependent changes in protein expression will contribute to a distinct temporal profile of statin effects. The consequences of short-term treatment of cells *in vitro*, a commonly used experimental model, may not reflect the consequences of chronic treatment. Furthermore, study of isolated cells removes any paracrine influences present in the intact heart. Together, this highlights the importance of using chronic *in vivo* statin treatment models to understand statins’ effects in the clinical setting.

In the main, work concerned with the direct effect of statins on the heart has focused on the drug’s ability to limit remodeling following ischemia-reperfusion (I-R) (Bauersachs et al., 2001; Verma et al., 2004; Di Napoli et al., 2005; Ye et al., 2008). These studies have shown that acute statin treatment *in vitro* increases Akt activation and NO production in human ventricular myocytes following hypoxia-reoxygenation protocols (Verma et al., 2004). In rat, chronic statin treatment *in vivo* has been shown to increase myocardial eNOS expression following (local/global) I-R protocols (Bauersachs et al., 2001; Di Napoli et al., 2005). Of note, the impact of chronic statin treatment *in vivo* on NO pathways in the healthy heart and the impact that this has on myocyte function have not been studied to date. These are important considerations as statins are prescribed to those without established cardiac disease.

Therefore, the aim of the present study was to describe the impact of chronic statin treatment *in vivo* on the contractile function of the ventricular myocyte and identify the mechanisms responsible. Using a rodent model, our data show no evidence of myocardial toxicity, but reveal an increase in myocyte lusitropy (independent of changes in Ca^2+^ transient decay) which can be ascribed to phosphorylation of the myofilament protein troponin I (TnI) and linked to both cholesterol-dependent and -independent modulation of NO production. Parallel changes were observed in myocardial samples from patients taking statins. Such effects at the level of the myocyte should prove beneficial, promoting diastolic function of the heart.

## Materials and Methods

### Ethics Statement

Human right atrial samples were obtained from male patients without a diagnosis of diabetes undergoing coronary artery by-pass surgery at the Leeds General Infirmary. All patients gave written informed consent. Data were anonymized. This study was approved by the Local Research Ethics committee (01/040, to April 2016) and complies with the principles outlined in the Declaration of Helsinki. Most atrial muscle samples (from both control and statin-treated individuals) contained some fatty tissue which was removed before processing.

All animal experimentation was approved by the local Animal Welfare and Ethical Review committee and carried out in accordance with the Directive 2010/63/EU of the European Parliament. Adult male Wistar rats were group housed with 12 h light/dark cycles and free access to food and water. For 5 days prior to treatment, animals were acclimatized to reversed 12 h light/dark cycles, so that dosing could be performed at the beginning of the dark cycle, to coincide with peak HMG-CoA reductase activity (Easom and Zammit, 1984). Rats were administered 40 mg kg^-1^ simvastatin daily for 14 days by oral gavage of a 4 mg ml^-1^ simvastatin saline suspension. Control animals received equivalent volumes of saline by the same route. In the rat statins are generally given by oral gavage, to mimic the situation in the clinic whereby the inactive simvastatin lactone is hydrolyzed in the liver to the open acid active form of the drug (Gazzerro et al., 2012), at doses between 10 and 60 mg/kg/day (e.g., Delbosc et al., 2002; Baytan et al., 2008; Renno et al., 2015).

### Modified Langendorff Procedure

To reduce animal use, a method was deployed which permits isolation of cardiomyocytes and processing of whole muscle from the same heart (Macdougall and Calaghan, 2013). Dissociated myocytes were used in shortening/[Ca^2+^]_i_ experiments. Myocardial homogenates were used for sucrose density gradient fractionation, western blotting and measurement of NO metabolites.

### Shortening and [Ca^2+^]_i_

Shortening and [Ca^2+^]_i_ were measured in fura-2 loaded myocytes field-stimulated at 0.5 Hz (22–24°C) in HEPES-based physiological solution containing (mM): NaCl 137; KCl 5.4; NaH_2_PO_4_ 0.33; MgCl_2_ 0.5; HEPES 5; glucose 5.5; CaCl_2_ 1 (pH 7.4) (Cairn Research Optoscan monochromator; Ionoptix Cell Contractility System). Parameters were measured under basal conditions and following selective stimulation of β1 or β2 adrenoceptors (ARs). Recordings were taken at the peak steady-state response (≈5 min) following application of agonist. Selective β1-AR stimulation was achieved with 100 nM isoprenaline in the presence of 100 nM ICI 118,551; selective β2-AR stimulation was achieved with 10 μM zinterol in the presence of 300 nM CGP20712A.

### Western Blotting

Protein expression in myocardial homogenates was measured by Western blotting as described in (Calaghan et al., 1998). For unfractionated samples, protein expression was normalized to GAPDH. For measurement of phosphorylated proteins (eNOS, TnI, VASP), samples for total and phospho-proteins were run on separate gels to avoid the interference which may arise following a stripping protocol. For rodent samples (for VASP, Ser^239^ VASP) and all human atrial samples, where every control and statin sample could not be included on the same gel, a standard sample (SS) was included on each gel to allow inter-gel comparisons. For fractionated samples, equal volumes of fractions were loaded onto SDS-gels and band density normalized to the sum of the band density in all fractions.

### Sucrose Density Gradient Fractionation

Myocardium was fractionated using detergent-free methods as described previously (Calaghan et al., 2008). Samples were homogenized (Ultra-Turrax T8; Ika), then sonicated (Vibra Cell; Sonics) three times each for 20 s at full power in 500 mM Na_2_CO_3_ (pH 11.0) containing 0.5 mM EDTA and 1% protease inhibitor cocktail (Sigma). Approximately, 2 ml of homogenate was mixed with an equal volume of 90% sucrose in MES-buffered saline (25 mM MES, 150 mM NaCl, 2 mM EDTA, pH 6.5) to form a 45% sucrose solution. A discontinuous sucrose gradient was created by layering on to this a further 4 ml each of 35 and 5% sucrose solution (MES-buffered saline with 250 mM Na_2_CO_3_). Gradients were centrifuged for 17 h at 280,000 *g* (Beckman SW40Ti rotor) at 4°C. A total of 12 fractions (each 1 ml) were collected following centrifugation.

### Measurement of NO Metabolites

Quantification of nitrite and nitrate levels in myocardial homogenates was achieved using a method outlined by Nussler et al. (2006). Freshly prepared homogenates were ultra-filtrated using Centrisart 1 columns with a molecular weight cut-off of 10 kDa (Sartorius #12329E). Nitrate pools from resulting ultra-filtrates were converted to nitrite with nitrate reductase and the total nitrite concentration of each sample was determined using 2,3-diaminonapthalene, as described (Nussler et al., 2006).

### Reagent Supplies

All reagents were from Sigma Aldrich unless otherwise stated. Antibodies used for Western blotting: Cav1 #610406 (1:5000), Cav 3 #610420 (1:5000), eNOS #610296 (1:1000), phospho- Ser^1177^- eNOS # 612392 (1:1000) (BD Biosciences), troponin I (TnI) #4002 (1:1000), Ser^23,24^ phospho-TnI #4004 (1:1000), VASP #3112 (1:1000), Ser^239^ phospho-VASP #3114 (1:1000) (Cell Signalling Technology), and GAPDH #G9545 (1:50000) (Sigma).

### Statistics

Results are expressed as mean ± SEM of *n* (cells) and N (animals/patients) observations. Statistical analysis was performed using the Student’s *t*-test.

## Results

In order to select a clinically relevant simvastatin dose, we considered serum levels of the drug and its IC_50_; a daily dose of simvastatin (40 mg/kg) was used to give peak plasma levels between 5 and 10 times the IC_50_ in the rat (see Germershausen et al., 1989; Masters et al., 1995). This is higher than the dose used in man (up to 80 mg daily; i.e., 1.1 mg/kg for a 70 kg patient) because the hepatic activity of HMG CoA reductase has been estimated to be up to 10 times higher in rat than man (Dansette et al., 2000). Animals were treated with statins for 2 weeks to allow sufficient time for compensatory changes in elements of the cholesterol homeostasis pathways to occur.

### Simvastatin Treatment in the Rat Selectively Increases Ventricular Myocyte Lusitropy

**FIGURE 1:**
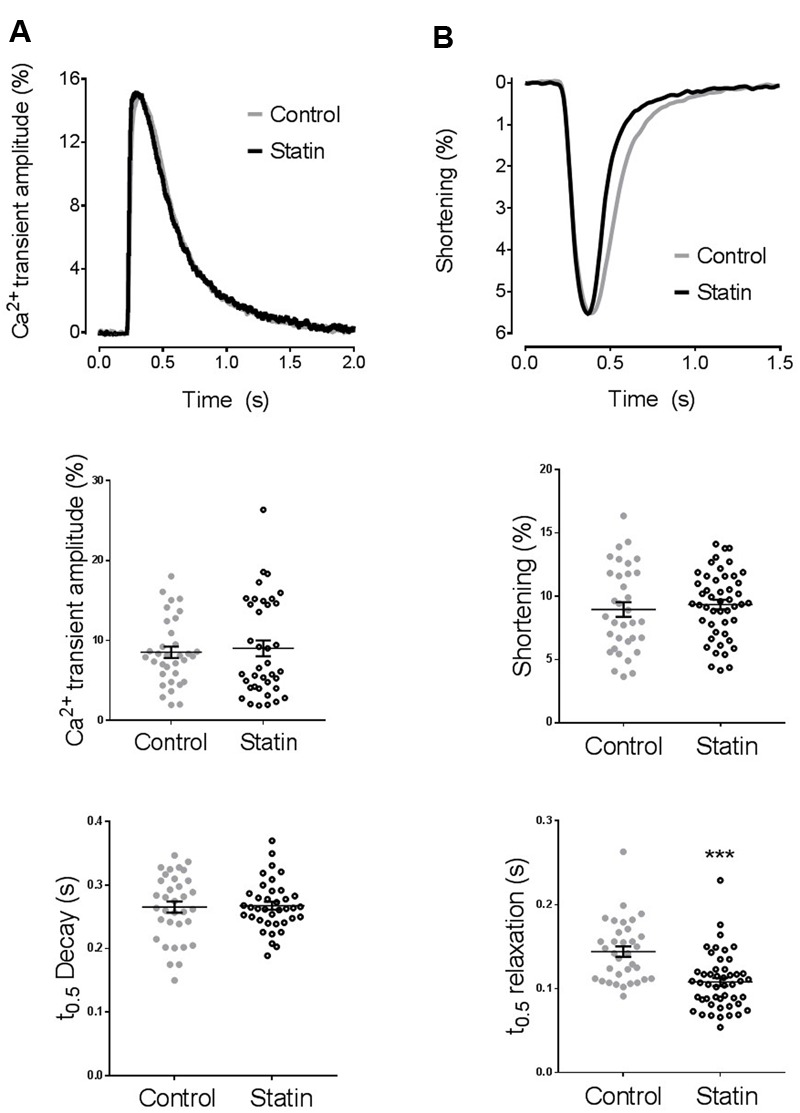
**The effect of simvastatin treatment on [Ca^2+^]_i_ transient and shortening. (A)** Representative traces and scatter plots of amplitude and kinetics of the [Ca^2+^]_i_ transient. Transient amplitude is expressed as % change in fura-2 ratio units (RU) over baseline. There was no difference in diastolic [Ca^2+^]_i_ between groups (0.967 ± 0.008 vs. 0.976 ± 0.003 RU in cells from control and statin-treated animals respectively). *n* = 34–35 cells from 7 to 8 animals per group. **(B)** Representative traces and scatter plot of amplitude and kinetics of shortening. Shortening amplitude is expressed as % of resting cell length. There was no difference in resting cell length between groups (110 ± 2 vs. 110 ± 2 μm in cells from control and statin-treated animals respectively). Plots show mean ± SEM from *n* = 34–48 myocytes from 7 to 8 animals per group. ^∗∗∗^*P* < 0.001 vs. control group, Student’s *t*-test.

We began by looking at the consequences of statin treatment for isolated ventricular myocyte function (**Figure 1**). Simvastatin had no effect on the amplitude of the [Ca^2+^]_i_ transient or shortening. The rate of decay of the [Ca^2+^]_i_ transient was similar in cells from statin treated animals and controls, however, there was a marked hastening of the rate of relaxation (*P* < 0.001) with statin treatment, as indexed by the time to half (*t*_0.5_) relaxation (0.108 ± 0.005 vs. 0.144 ± 0.006 s; *n* = 48/34 cells, *N* = 8/7 animals in statin/control groups).

### Simvastatin Treatment Increases Phosphorylation of Troponin I

**FIGURE 2:**
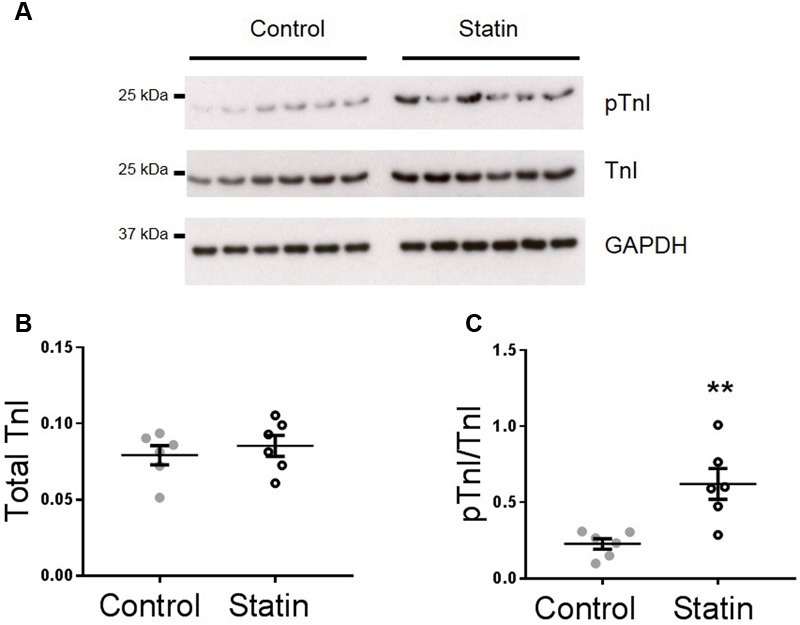
**Simvastatin treatment increases phosphorylation of troponin I (TnI).** Representative immunoblots **(A)** and scatter plots showing myocardial levels of total troponin I **(B)** and Ser^23/24^ phosphorylated TnI (pTnI) **(C)** in control and statin-treated animals. Total TnI is normalized to GADPH, pTnI is normalized to total TnI. Plots show mean ± SEM from *N* = 6 animals per group. ^∗∗^*P* < 0.01 vs. control group, Student’s *t*-test.

Increased lusitropy in the absence of a corresponding change in [Ca^2+^]_i_ transient decay is consistent with increased phosphorylation of TnI which enhances the off-rate of Ca^2+^ from TnC (Zhang et al., 1995). Indeed, there was a 2.7-fold increase in phosphorylation of TnI at Ser^23/24^ (*P* < 0.01) in myocardium from statin-treated rats compared with controls [0.62 ± 0.1 vs. 0.23 ± 0.03 arbitrary units (AUs); *N* = 6], in the absence of a change in TnI expression (**Figure 2**).

### Simvastatin Treatment and eNOS Activity

**FIGURE 3:**
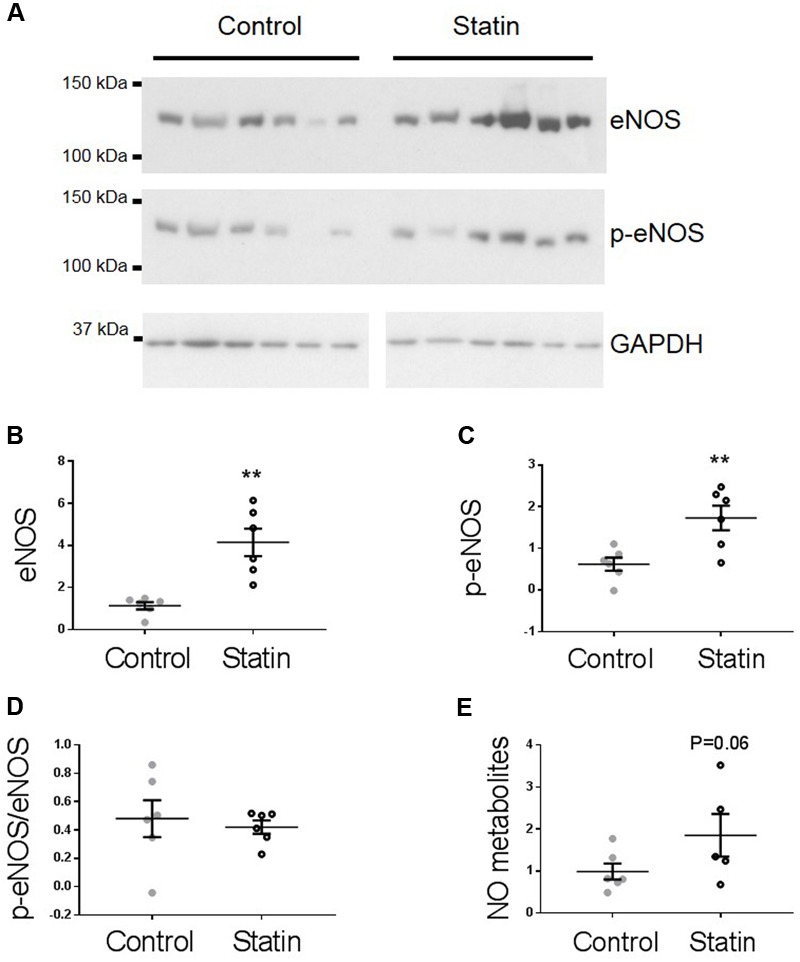
**Simvastatin increases eNOS expression and phosphorylation.** Representative immunoblots **(A)** and scatter plots showing myocardial levels of eNOS **(B)** and Ser^1177^ phosphorylated eNOS (p-eNOS, **C**) normalized to GAPDH. **(D)** The proportion of total eNOS phosphorylated at Ser^1177^ was not different between groups. **(E)** NO metabolites (nitrate + nitrite) measured by a fluorimetric assay. Plots show mean ± SEM from *N* = 6 animals per group. ^∗∗^*P* < 0.01 vs. control group, Student’s *t*-test.

**FIGURE 4:**
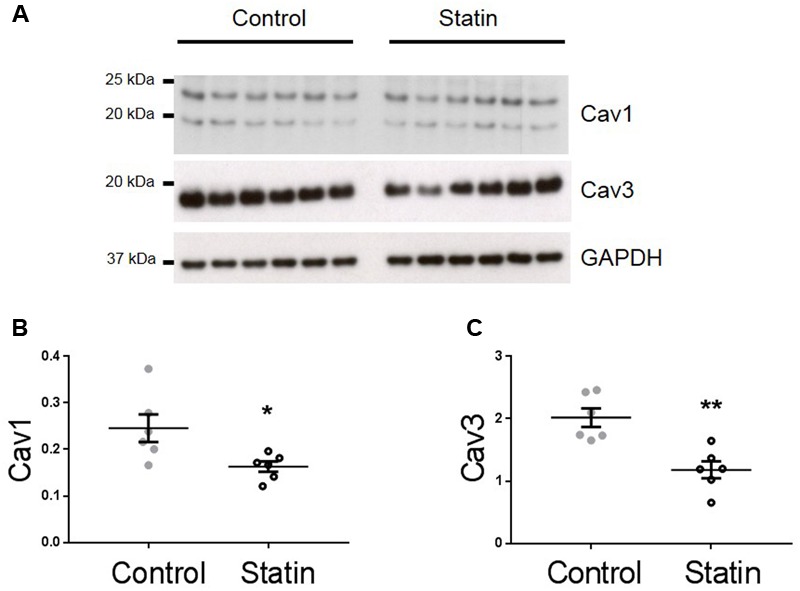
**Simvastatin reduces expression of caveolins 1 and 3.** Representative immunoblots **(A)** and scatter plots showing myocardial expression of Cav1 α (upper band) and β isoforms **(B)** and Cav3 **(C)**. Plots show mean ± SEM from *N* = 6 animals per group. ^∗^*P* < 0.05, ^∗∗^*P* < 0.01 vs. control group, Student’s *t*-test.

TnI is phosphorylated at Ser^23/24^ by both protein kinase (PK) A and PKG, and PKG is a likely mediator of this phosphorylation as its activity is stimulated by NO and NO bioavailability is increased by *in vitro* statin treatment in both endothelial cells and cardiac myocytes (Feron et al., 2001; Pugh et al., 2014). Therefore, we next measured indices of NO production: eNOS expression and phosphorylation, and NO metabolites. As shown in **Figure 3**, simvastatin treatment increased the expression of eNOS (4.15 ± 0.65 vs. 1.14 ± 0.17 AU; *N* = 6) and Ser^1177^-phosphorylated eNOS (1.73 ± 0.29 vs. 0.62 ± 0.16 AU; *N* = 6), although the proportion of total eNOS phosphorylated was unchanged. There was a trend (*P* = 0.06) for an increase in NO metabolites (nitrates and nitrites) with statin treatment (1.85 ± 0.51 vs. 0.99 ± 0.19 AU; *N* = 5/6). In addition to increased eNOS expression and phosphorylation, NO production can be enhanced by reduced expression of caveolin (Cav), the constitutive inhibitor of NOS. Acute *in vitro* statin treatment reduces Cav3 expression in the adult cardiac myocyte (Pugh et al., 2014). In the present study, chronic *in vivo* statin treatment reduced myocardial expression of both the muscle-specific Cav3 (1.18 ± 0.14 vs. 2.02 ± 0.15 AU; *N* = 6) and the ubiquitously expressed Cav1 (0.16 ± 0.01 vs. 0.25 ± 0.03 AU; *N* = 6) (*P* < 0.05, see **Figure 4**), which would be predicted to enhance eNOS activity. These are important findings because, although SRE control of Cav1 is established (Bist et al., 1997), there is no direct evidence of the same control for Cav3.

**FIGURE 5:**
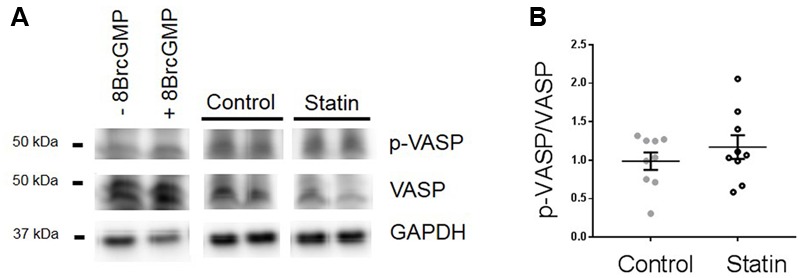
**Effect of simvastatin on Ser^239^-phosphorylated VASP, an index of PKG activity. (A)** Representative immunoblots showing Ser^239^-phosphorylated VASP and VASP expression in rat ventricular myocytes ± 8-Bromo cGMP (200 μM for 15 min, as a positive control for PKG activity) and myocardial homogenates from control and statin-treated animals. **(B**) Ratio of p-VASP to VASP. Plot shows mean ± SEM of *N* = 9 animals per group.

We were not able to index PKG activity directly due to limitations of tissue supply (hearts were used for both myocyte and myocardial homogenate preparations). Instead we used an alternative means of assessing PKG activity through measurement of phosphorylation of vasodilator stimulated phosphoprotein (VASP) at Ser^239^, which is the site preferentially targeted by PKG. This approach is commonly employed to assess PKG activity in both myocyte and myocardial preparations (e.g., Sartoretto et al., 2009; van Heerebeek et al., 2012). As shown in **Figure 5**, there was a trend for an increase in the ratio of Ser^239^-phospho VASP:VASP in statin-treated samples (1.18 ± 0.15 vs. 0.99 ± 0.11 ratio units; *N* = 9) consistent with NO-dependent stimulation of PKG.

### Simvastatin Treatment and Serum/Myocardial Cholesterol

**FIGURE 6:**
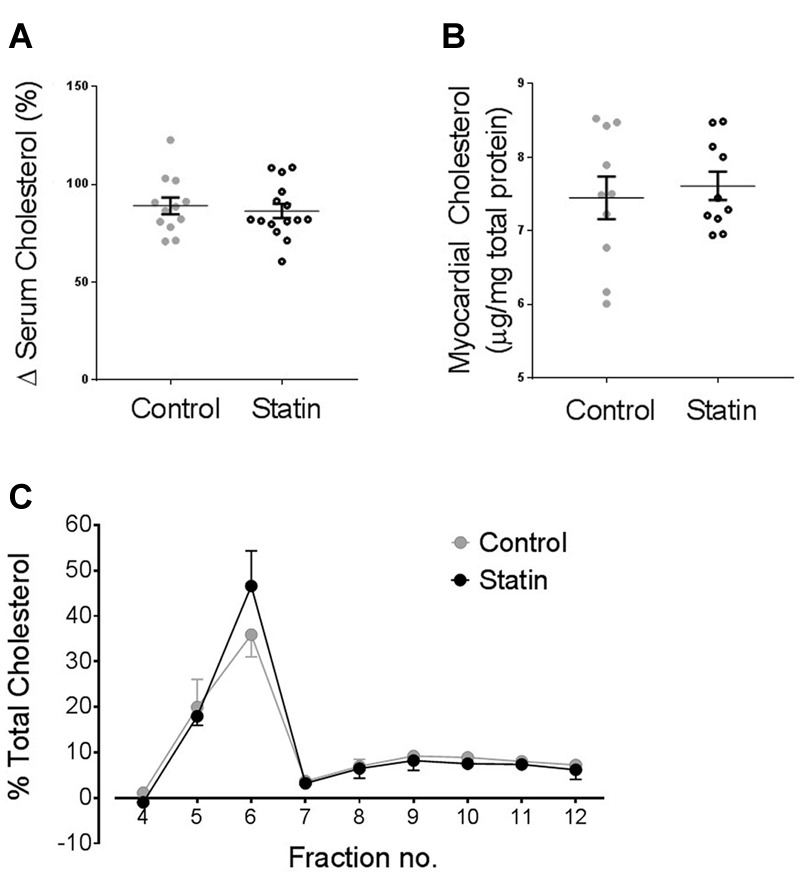
**Statin treatment for 2 weeks has no impact on serum or myocardial cholesterol levels. (A)** Serum cholesterol expressed as a % change from values on day 0 (*N* = 12–15 animals). **(B)** Myocardial cholesterol, normalized to myocardial total protein (*N* = 10 animals). **(C)** Myocardial cholesterol following sucrose gradient fractionation, values are expressed as a % of total cholesterol across all fractions (*N* = 3 animals).

Statin treatment over a 2 week period had no impact on serum or cholesterol (**Figure 6A**) which is consistent with other studies using normocholesterolemic rodents (Chen et al., 2003; Whitehead et al., 2015). We have previously shown reduced myocyte cholesterol levels in adult cardiac cells acutely treated with simvastatin for 2 days *in vitro*, consistent with reduced *de novo* myocyte cholesterol synthesis (Pugh et al., 2014). However, with prolonged statin treatment, as used in the present study, we would predict that SRE-dependent changes in cholesterol homeostatic proteins, including Cav, would act to return myocardial cholesterol to normal levels. This prediction is supported by data showing no difference in myocardial cholesterol between control and statin-treated animals (**Figure 6B**). There was also no significant difference in the distribution of cholesterol between membrane fractions (**Figure 6C**).

### Effect of Statin on β-AR Responsiveness

**FIGURE 7:**
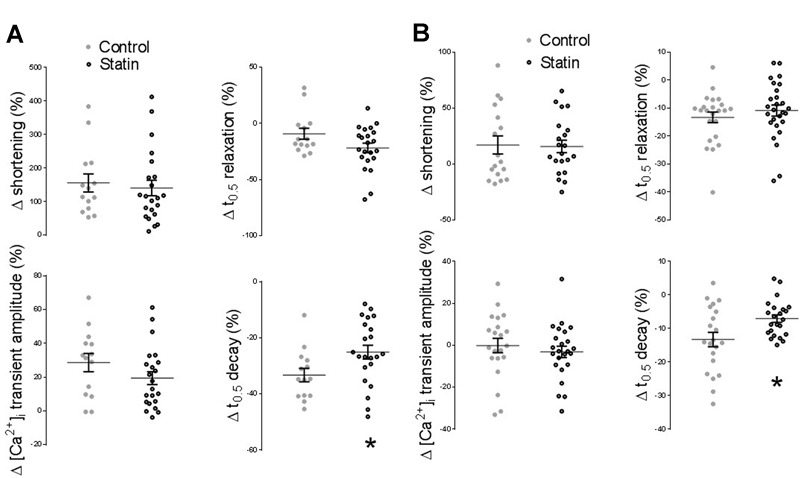
**Effect of statin treatment on the response to β-adrenoceptor (AR) stimulation. (A)** Percentage change from baseline in amplitude/kinetics of the [Ca^2+^]_i_ transient and shortening in response to β1-AR stimulation with 100 nM isoprenaline in the presence of 100 nM ICI 118,551 (*n* = 14–22 cells from 3 to 4 animals per group). **(B)** Percentage change from baseline in amplitude/kinetics of the [Ca^2+^]_i_ transient and shortening in response to β2-AR stimulation with 10 μM zinterol in the presence of 300 nM CGP20712A (*n* = 23–28 cells from 4 animals per group). Plots show mean ± SEM. ^∗^*P* < 0.05 vs. saline-treated group, Student’s *t*-test.

Acute cholesterol depletion of adult cardiac myocytes with methyl-β-cyclodextrin (which displaces Cav3 from caveolar domains) has been shown to increase the sensitivity of β1-adrenoceptor (AR) responses and to markedly enhance β2-AR responsiveness (Agarwal et al., 2011; Macdougall et al., 2012). Cardiac myocytes treated with simvastatin *in vitro* over 2 days show enhanced inotropic responses to β2-AR stimulation (with no change in β1-AR responses), in association with reduced levels of Cav3 and cholesterol (Pugh et al., 2014). An important question is whether statin treatment for 2 weeks *in vivo*, which reduces Cav1/3 expression without affecting myocardial cholesterol, modulates β-AR responsiveness. There was no significant effect of simvastatin treatment on the inotropic or lusitropic response to β1- or β2-AR stimulation (see **Figure 7**). However, statin treatment attenuated (*P* < 0.05) the increased rate of [Ca^2+^]_i_ transient decay (indexed by % change in *t*_0.5_ transient decay) which was seen with both β1-AR stimulation (-25 ± 2 vs. -33 ± 2%; *n* = 22/14 cells) and β2-AR stimulation (-7.1 ± 1.1 vs. -13 ± 2%; *n* = 23/21 cells, *N* = 4/3 animals).

### Are Observations Made in Rats Reflective of the Human Population?

**FIGURE 8:**
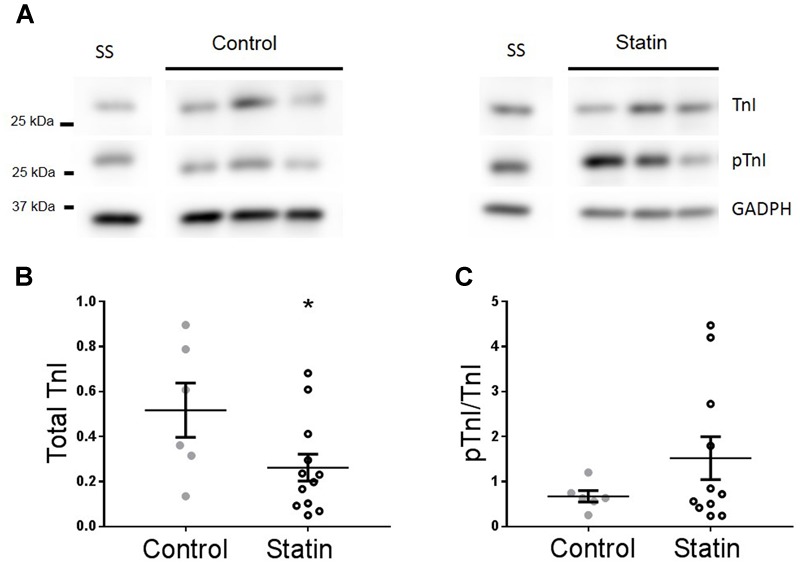
**Effect of statin treatment on TnI and Ser^23/24^ phosphorylated TnI (pTnI) in human myocardium.** Representative immunoblots **(A)** and scatter plots showing expression of total TnI normalized to GAPDH **(B)** and pTnI normalized to total TnI **(C)** in atrial muscle from statin-treated male patients (*N* = 14). Patients not taking statins formed the control group (*N* = 6). SS = standard sample to allow inter-gel comparisons (see Materials and Methods, Western blotting). Plots show mean ± SEM. ^∗^*P* < 0.05 vs. control group (Student’s *t*-test).

**FIGURE 9:**
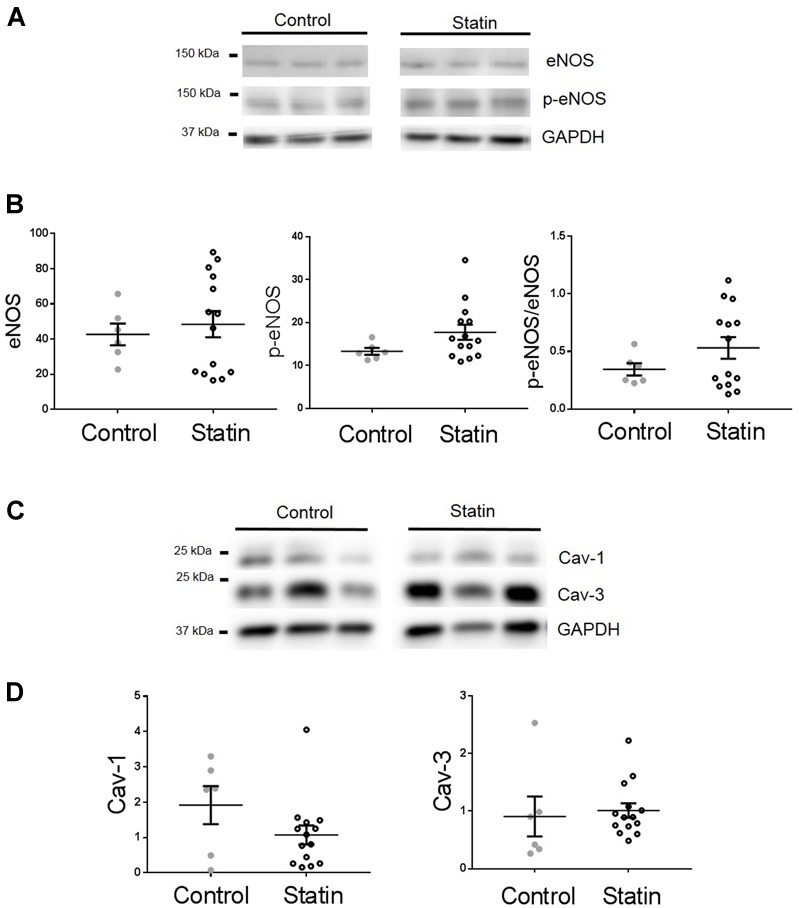
**Effect of statin treatment on proteins that regulate NO production in human myocardium.** Representative immunoblots from the same gel and scatter plots showing expression of eNOS and Ser^1177^ phosphorylated eNOS (p-eNOS) **(A,B)**, Cav-1 and Cav-3 **(C,D)** in atrial muscle from statin-treated male patients (*N* = 14). Male patients not taking statins formed the control group (*N* = 6). Plots show mean ± SEM.

In order to establish whether observed changes in TnI, eNOS, and Cav in our rodent model are of clinical relevance, we obtained samples (*n* = 22) of human (atrial) myocardium from male patients undergoing coronary artery bypass surgery, the majority of whom were taking statins at the time of surgery. See **Table 1** for patient data. There was no difference in age between control and statin groups, and those with a diagnosis of diabetes were excluded from the study (as diabetes has a myriad of cardiac effects including changes in Cav expression) (Murfitt et al., 2015). Key changes in protein expression/phosphorylation with statin treatment in the rat were mirrored in human samples, although possibly because of the small sample size these did not attain statistical significance. Sample size is limited by human tissue availability; it is particularly difficult to find patients undergoing by-pass surgery who are *not* prescribed statins (limited to *N* = 6 in our study). Levels of phosphorylated TnI (as a proportion of total TnI) were increased 2.2-fold (*P* = 0.2) with statin treatment (1.52 ± 0.48 vs. 0.67 ± 0.13 AU; *N* = 14/6) (**Figure 8**). Whilst eNOS levels were similar between groups, there was a trend for an increase (*P* = 0.1) in phosphorylated eNOS in the statin group (17.7 ± 1.7 vs. 13.3 ± 0.8 AU; *N* = 14/6). Cav-1 levels were reduced by ≈50% (*P* = 0.1) in the statin-treated group (1.07 ± 0.27 vs. 1.92 ± 0.54 AU; *N* = 14/6) whereas Cav-3 expression was similar between groups (**Figure 9**). Thus human data are in general agreement with our findings from a rodent model that chronic statin treatment enhances TnI phosphorylation which could be ascribed in part to enhanced NOS activity.

**Table 1:** Details of male patients from which right atrial samples were obtained.

Age	Statin	Dose (mg/day)
65	–	–
74	–	–
54	–	–
30	–	–
75	–	–
65	–	–
64	Atorvastatin	10
71	Simvastatin	20
60	Atorvastatin	20
79	Atorvastatin	20
68	Pravastatin	40
72	Atorvastatin	40
49	Rosuvastatin	10
69	Atorvastatin	40
71	Atorvastatin	40
55	Atorvastatin	80
72	Atorvastatin	80
50	Atorvastatin	80
65	Atorvastatin	80
68	Atorvastatin	80

*All patients were undergoing coronary artery by-pass surgery. None had received a diagnosis of diabetes. There was no difference in age between the control (61 ± 7, *n* = 6) and statin-treated (65 ± 2, *n* = 14) groups (*P* > 0.05).*

## Discussion

Here, we show a pleiotropic effect of statin treatment that would be of benefit to cardiac function. Simvastatin treatment increases the rate of relaxation of the cardiac myocyte, consistent with a NO-dependent modulation of TnI phosphorylation mediated via PKG.

### Basal Myocyte Function and β-AR Responsiveness

We have previously shown that acute simvastatin treatment of cardiac myocytes *in vitro* has lusitropic effects, but these were associated with corresponding changes in the [Ca^2+^]_i_ transient which suggests an alternative mechanism. A few studies have examined the impact of chronic statin treatment *in vivo* on basal cardiac function. In common with our own findings, these studies report that statins have no impact on indices of contractility. In the rat, a 2 week treatment with atorvastatin had no effect on the magnitude of contraction in isolated atrial preparations (Schmechel et al., 2009) and after 10 weeks, no alternations in cardiac function (ejection fraction, maximum rate of pressure change) were detected by echocardiography (Yao et al., 2009). With reference to improved diastolic function, a 6 month treatment with rosuvastatin has been shown to significantly reduce the Tei index, a parameter which incorporates myocardial relaxation time, in patients without evidence of coronary artery disease (Talini et al., 2008). By contrast, other work has shown that 70% of naïve patients without overt cardiac dysfunction who were given atorvastatin for up to 6 months developed worsening diastolic function (defined as a 10% change in three echocardiographic indices of diastolic performance) (Silver et al., 2004). In both these studies, individuals were hypercholesterolemic, so the impact of statin treatment on serum cholesterol must be considered.

Treatment of rats for 2 weeks with 40 mg/kg simvastatin has no impact on the inotropic or lusitropic response of isolated ventricular myocytes to stimulation of β1- and β2-ARs. However, the fact that lusitropy is unchanged whilst [Ca^2+^]_i_ transient decay is slowed could suggest a minor potentiation of lusitropic responses to β-AR with statin treatment (through enhanced phosphorylation of TnI) which is masked by changes in the [Ca^2+^]_i_ transient. The lack of impact of statin treatment on inotropic responses to β2-AR stimulation contrasts with the effect of *in vitro* statin treatment which markedly enhances this (Pugh et al., 2014). We suggest that the ability of short-term statin treatment to reduce myocyte cholesterol underlies its impact on β2-AR stimulation, as its effects are mirrored by acute cholesterol depletion of the cardiac cell with methyl β cyclodextrin (Macdougall et al., 2012). Reduction in membrane cholesterol may be essential to cause sufficient disruption of the caveolar microdomain, which normally facilitates coupling between the β2-AR and the inhibitory G protein, G_αi_ (see, Macdougall et al., 2012). It is interesting to note that the ≈40% loss of both Cav isoforms is not associated with major cardiac dysfunction and, taken in the context of treatments which disrupt the caveolar domain by cholesterol depletion or Cav knockout (Galbiati et al., 2001), may suggest that the caveolae contain a functional excess of Cav protein. Other data for effects of *in vivo* statin treatment on β-AR responsiveness are sparse. A minor decrease in the inotropic response of atrial preparations to non-selective β-AR stimulation with isoprenaline has been reported in rats treated for 2 weeks with atorvastatin (Schmechel et al., 2009).

### NO Bioavailability

Our data suggest that statins have multiple effects which contribute to elevated NO bioavailability. In the rat, statin treatment increases the expression of myocardial eNOS and phospho-eNOS and reduces expression of two of its inhibitory mediators Cav1 and Cav3. These data accord with many other reports in the literature of statins’ ability to increase NO in various cells types. In the intact heart, the functional relevance of increased NO with *in vivo* statin treatment has been demonstrated. For example, in pigs treated for 5 days with rosuvastatin, cardioprotective effects (reduced infarct size and improved mechanical function after I-R) were absent when a NOS inhibitor was administered concurrently with statins (Bulhak et al., 2005). In this case, increased eNOS expression was not responsible for enhanced NO bioavailability.

The source of enriched eNOS protein and NO production is an important question. eNOS is expressed in endothelial cells and cardiac myocytes, and both cell types are present in the myocardial samples used for protein expression data presented in **Figure 3**. The ability of statins to increase endothelial cell NO bioavailability is well-established (Laufs et al., 1998; Kureishi et al., 2000; Feron et al., 2001). However, as lusitropic effects of statin treatment are observed in cardiac myocytes this suggests that myocyte NO production is targeted by statins, as one might predict that paracrine (endothelial) NO effects would not persist in the isolated myocyte. This does not preclude a contribution from endothelial cells in the intact heart; the functional effects we have documented may well be underestimated if this is the case.

### Caveolin Expression

Sterol regulatory element transcriptional control acts to preserve cellular cholesterol levels following inhibition of *de novo* cholesterol synthesis. Caveolae are sites of cholesterol efflux (Fielding and Fielding, 1995). Recent data (from non-cardiac cells) suggest that caveolin regulates cholesterol efflux to HDL through direct interaction with the ATP binding cassette transporter G1 (ABCG1) (Gu et al., 2014). SRE transcriptional control of Cav1 has been established (Bist et al., 1997) suggesting that caveolin is implicated in cellular cholesterol homeostasis. Our data support the concept of SRE control of Cav1 and Cav3 transcription. We saw a ≈40% reduction in expression of both Cav isoforms in the statin group. Myocardial cholesterol is unchanged at this time point, consistent with SRE-dependent regulation of cellular cholesterol. Comparison with data from *in vitro* statin treatment for 2 days (Pugh et al., 2014) supports the concept that, whilst myocardial cholesterol initially falls with statin treatment, it is normalized after >2 days, in part through down-regulation of Cav which normally contributes to cellular cholesterol efflux. Of note, most eNOS is immunoprecipitated by antibodies to Cav1 in endothelial cells and by antibodies to Cav3 in cardiac myocytes (Feron et al., 1996). Thus our rodent data are consistent with the view that eNOS activity is enhanced in endothelial cells and myocytes by reductions in Cav1 and Cav3 expression respectively.

### TnI Phosphorylation

In the cardiac myocyte, NO is the main activator of soluble guanyl cyclase (sGC). The resulting elevation of cGMP has several consequences, one of which is stimulation of protein kinase G. Troponin I is a target of PKG in the cardiac myocyte (Yuasa et al., 1999); its phosphorylation at Ser^23/24^ increases the off-rate of Ca^2+^ from TnC and hastens relaxation. NO donors and elevation of cGMP have similar effects on relaxation rate in ferret papillary muscle (Smith et al., 1991). We did not detect any decrease in contraction amplitude in cells from statin treated animals which might be predicted as a result of decreased TnC Ca^2+^ affinity; this suggests some additional compensatory effects of statins. Phospholamban is another target of PKG, but the lack of increase in [Ca^2+^]_i_ transient amplitude or kinetics with statin treatment, suggests that compartmentation of cGMP signaling limits the targets of PKG in this case (see Castro et al., 2006). Of note, increased myocardial cGMP has been linked with cardioprotective effects during episodes of I-R (Bice et al., 2014) and PKG has been proposed as one of the salvage kinases that protects the heart from I-R damage (Burley et al., 2007). Support for an inverse correlation between cholesterol and PKG activity comes from data showing that rats fed a high cholesterol diet have decreased myocardial activity of PKG, as assessed by phosphorylation of TnI (Giricz et al., 2009), although in this study the mechanism that links altered cholesterol with PKG activity was not established.

### Human Data

Human samples come from a heterogeneous population (in terms of disease severity, medication, length of statin exposure, etc.) and there is limited availability of ‘control’ samples as most patients undergoing cardiac surgery will be taking these drugs. This contrasts with the well-controlled experiments possible using a rodent model. Despite the limitations of working with human tissue, the fact that our data from these samples show similarities to findings in rodent muscle (in terms of clear trends for changes in phosphorylated TnI, phosphorylated eNOS and Cav1) is persuasive.

A significant reduction in myocardial levels of TnI was observed in samples from individuals taking statins which was absent in the rodent model. Reduced myocardial TnI could be explained by a loss of protein from damaged muscle, perhaps because of more severe disease in this patient group; TnI loss has been reported in a variety of cardiac diseases, including post-infarction remodeling (Ricchiuti et al., 1997). If this is the case, it should be noted that statins are prescribed to those without established cardiac disease, therefore loss of TnI would not be a universal finding in all who take these drugs. Another possibility to explain TnI loss is statin-linked myopathy, similar to that reported in skeletal muscle (Bruckert et al., 2005), however, there are no reports of cardiac myopathy with statin treatment (see, Bouitbir et al., 2011) and we also saw no effect of statin treatment on total TnI expression in our rodent model. Overall, the important finding from atria from statin-treated individuals is a trend for an increase in the proportion of TnI which is phosphorylated, consistent with data in the rat.

Although eNOS expression was similar in control and statin groups, there was a trend for increased eNOS phosphorylation and reduced Cav1 expression in the statin-treated group. This correlates well with established sensitivity of mechanisms of altered NO production in other tissues. In endothelial cells it is Akt-dependent eNOS phosphorylation and Cav1 expression that are most sensitive to statins effects (Feron et al., 2001; Urbich et al., 2002). For example, reduced Cav 1 abundance is observed at 1000 times lower concentrations of atorvastatin than promoted increased eNOS expression. This suggests that phosphorylation of eNOS and depletion of Cav are likely to be the main mechanisms for statin-induced alterations in NO *in vivo*. Of note, if control of endothelial and cardiac myocyte eNOS in the rat heart is mirrored in the human heart, this implies that the primary Cav dependent impact of statin treatment could be on endothelial cell NO production, but this is likely to have paracrine influences on the myocyte *in situ* via PKG activation and TnI phosphorylation.

## Conclusion

We have shown for the first time that statin treatment *in vivo* has effects on cardiac myocyte relaxation, which are associated with changes which increase NO bioavailability and phosphorylation of the myofilament protein TnI. One limitation of the work is that it only shows association between aspects of contractile function, proteins which control NO production and TnI phosphorylation. Cause and effect has not been directly established. However, available literature for statin effects on NO, and NO effects on TnI phosphorylation provide strong support for the concept. These data suggest that autocrine and paracine effects of NO released within the myocardium will improve cardiac filling *per se*. Furthermore, the role of NO in cardioprotection suggests that this effect would have other benefits during times of cardiac stress (I-R). These data support the concept of an additional pleiotropic action of statins that improves cardiac function and cardioprotection even in individuals without established cardiac disease.

## Author Contributions

DM, SP designed work, acquired and analyzed and interpreted data, critically revised manuscript, approved final version, are accountable for all aspects of the work; HB, SL acquired and analyzed data, helped draft and revise manuscript, approved final version, are accountable for all aspects of the work; KP designed work, interpreted data, critically revised manuscript, approved final version, is accountable for all aspects of the work; SC conceived and designed work, interpreted data, wrote manuscript, approved final version, is accountable for all aspects of the work.

## Conflict of Interest Statement

The authors declare that the research was conducted in the absence of any commercial or financial relationships that could be construed as a potential conflict of interest. The reviewer GV and handling Editor declared their shared affiliation, and the handling Editor states that the process nevertheless met the standards of a fair and objective review.
